# Atypical Cause of Autoimmune Encephalitis: The Role of Mycoplasma pneumoniae in Status Epilepticus

**DOI:** 10.7759/cureus.84689

**Published:** 2025-05-23

**Authors:** Miles Horton, Mai-Linh Nguyen, Michelle Mateus Twitchell

**Affiliations:** 1 Medicine, Dr. Kiran C. Patel College of Osteopathic Medicine, Nova Southeastern University, Davie, USA; 2 Internal Medicine, Palmetto General Hospital, Miami, USA

**Keywords:** altered mental status, autoimmune encephalitis, drug-refractory seizures, mycoplasma, status epilepticus

## Abstract

Autoimmune encephalitis is a group of conditions characterized by a rapid onset of neurological symptoms, such as seizures and cognitive impairment, along with psychiatric disturbances such as behavioral changes and psychosis. Viral infections are common, well-documented triggers, but the role of bacterial pathogens, particularly *Mycoplasma pneumoniae*, remains poorly understood. *Mycoplasma pneumoniae* has been implicated in autoimmune encephalitis previously, but reported cases are rare. This report presents a case of *Mycoplasma*-induced seronegative autoimmune encephalitis in a previously healthy adult, highlighting the diagnostic challenges and the need for early recognition. The patient, a 37-year-old man with a past medical history of asthma, presented with encephalopathy and confusion following a fall in the shower a few hours previously. He then experienced refractory seizures necessitating intensive immunosuppressive and antimicrobial therapy. After a four-month hospital stay, primarily in the ICU, the patient returned to his baseline and was discharged home after four days without experiencing any new neurological deficits or seizures. Given the potential for delayed diagnosis and severe neurological complications, increased clinical awareness and further research are needed to improve diagnostic and therapeutic strategies for *Mycoplasma*-associated autoimmune encephalitis.

## Introduction

Autoimmune encephalitis refers to a group of inflammatory brain disorders caused by an abnormal immune response in which the body’s immune system mistakenly attacks components of the central nervous system. Symptoms can vary significantly at onset and range from mild cognitive disturbances to severe seizures, altered mental status, and psychiatric manifestations such as hallucinations or paranoia [1]. Because of its often severe clinical presentation and the possibility of full recovery with effective treatment, autoimmune encephalitis has gained significant attention in both clinical practice and in research. While a number of underlying triggers, including tumors, viral infections, and genetic variables, have been observed in causing autoimmune encephalitis, the role that bacterial infections play in autoimmune encephalitis is still poorly understood and rarely documented.

The underlying mechanism of autoimmune encephalitis is mediated through an immune response triggered by an infection or external factors. Inflammation and neural dysfunction arise when the immune system unintentionally attacks neurons or neuronal surface proteins, such as voltage-gated potassium channels or N-methyl-D-aspartate (NMDA) receptors [2]. In some cases, the immune system's cross-reaction between microbial antigens and neural tissue is caused by molecular mimicry, which results in an inadequate immune response [2]. The attack by the immune system can be mediated through an indirect process, with T-cell-mediated responses, or a direct process, with the generation of autoantibodies who do not directly bind to antigen [3]. 

Viral infections are recognized as potential triggers for autoimmune encephalitis, with *Herpes simplex* virus and *Varicella zoster* virus being the most commonly linked pathogens [3]. However, *Mycoplasma pneumoniae*, which is usually associated with atypical pneumonia and respiratory tract infections, has also been linked to the etiology of autoimmune encephalitis. Although the precise mechanism in which *Mycoplasma pneumoniae* causes autoimmune encephalitis is still unclear, it is believed that the pathogen's capacity to cause immunological dysregulation could result in the generation of autoantibodies that target the central nervous system, leading to severe neurological impairment [4]. Few case reports in literature have documented the correlation between *Mycoplasma pneumoniae* infection and autoimmune encephalitis. 

Diagnosis of autoimmune encephalitis requires a comprehensive yet timely approach, as early intervention is crucial for positive outcomes. The process typically involves a combination of clinical assessment, neuroimaging, cerebrospinal fluid (CSF) analysis, electroencephalography (EEG), and serological testing [5]. Brain MRI is often performed first, not only to rule out other possible diagnoses, but also to look for patterns that may suggest autoimmune encephalitis. Along with MRI, EEG can reveal nonspecific abnormalities such as diffuse slowing or epileptiform discharges, supporting the presence of encephalopathy, and is also used to monitor seizures, which are common. CSF analysis is typically the next step to test for specific neuronal autoantibodies, infections, cytology, and inflammatory markers. Importantly, treatment, particularly immunosuppressive therapy, should not be delayed while awaiting confirmatory results, as early intervention is critical in preventing permanent neurological damage. In cases where *Mycoplasma pneumoniae* is suspected as a trigger for autoimmune encephalitis, polymerase chain reaction (PCR) testing or serologic assays for recent infection should be obtained early.

Distinguishing *Mycoplasma*-induced autoimmune encephalitis from infectious encephalitis is challenging due to overlapping symptoms. Infectious encephalitis typically presents with acute onset, high fever, and a clear infectious prodrome, with CSF PCR detecting pathogens. In contrast, *Mycoplasma*-induced autoimmune encephalitis has a subacute onset, with prominent psychiatric symptoms or movement disorders, and fever may be absent. CSF in autoimmune cases often shows mild lymphocytic pleocytosis, normal glucose, and neuronal autoantibodies, with *Mycoplasma* PCR or serology indicating recent infection.

Treatment of autoimmune encephalitis involves immunosuppressive therapy to reduce inflammation. Intravenous immunoglobulin (IVIG; 2 g/kg over 2-5 days), plasmapheresis (5-10 sessions every other day), and corticosteroids (3-7-day course) are first line treatments to eliminate the autoantibodies from the bloodstream, and reduce inflammation in the brain [5]. Since these methods can help control seizures and prevent irreversible brain damage, they work best when started early. In situations of *Mycoplasma*-induced autoimmune encephalitis, the underlying infection may also be treated with antibiotics such doxycycline or macrolides [4]. *Mycoplasma*-induced autoimmune encephalitis can be a clinically challenging diagnosis. Thus, this can lead to delayed recognition and lead to potentially life-threatening manifestations such as frequent seizures, requiring acute neurological care. Ongoing research about this diagnosis can help with faster diagnoses and earlier interventions.

This report presents a rare case of *Mycoplasma*-induced autoimmune encephalitis, outlining the clinical presentation, overall diagnostic approach, and therapeutic interventions. It emphasizes the severity of this condition and its capacity to cause seizures and other neurological complications that require intensive care. This patient’s presentation initially mimicked other infectious or autoimmune disorders, underscoring the importance of considering less common infections as a differential diagnosis when autoimmune encephalitis is under consideration. Since early detection and treatment can greatly improve prognosis and lower the risk of long-term neurological complications, this case report emphasizes the need for greater awareness of *Mycoplasma*-induced autoimmune encephalitis as a differential diagnosis in order to reduce the severity of seizures and enhance recovery in these patients.

## Case presentation

**Figure 1:**
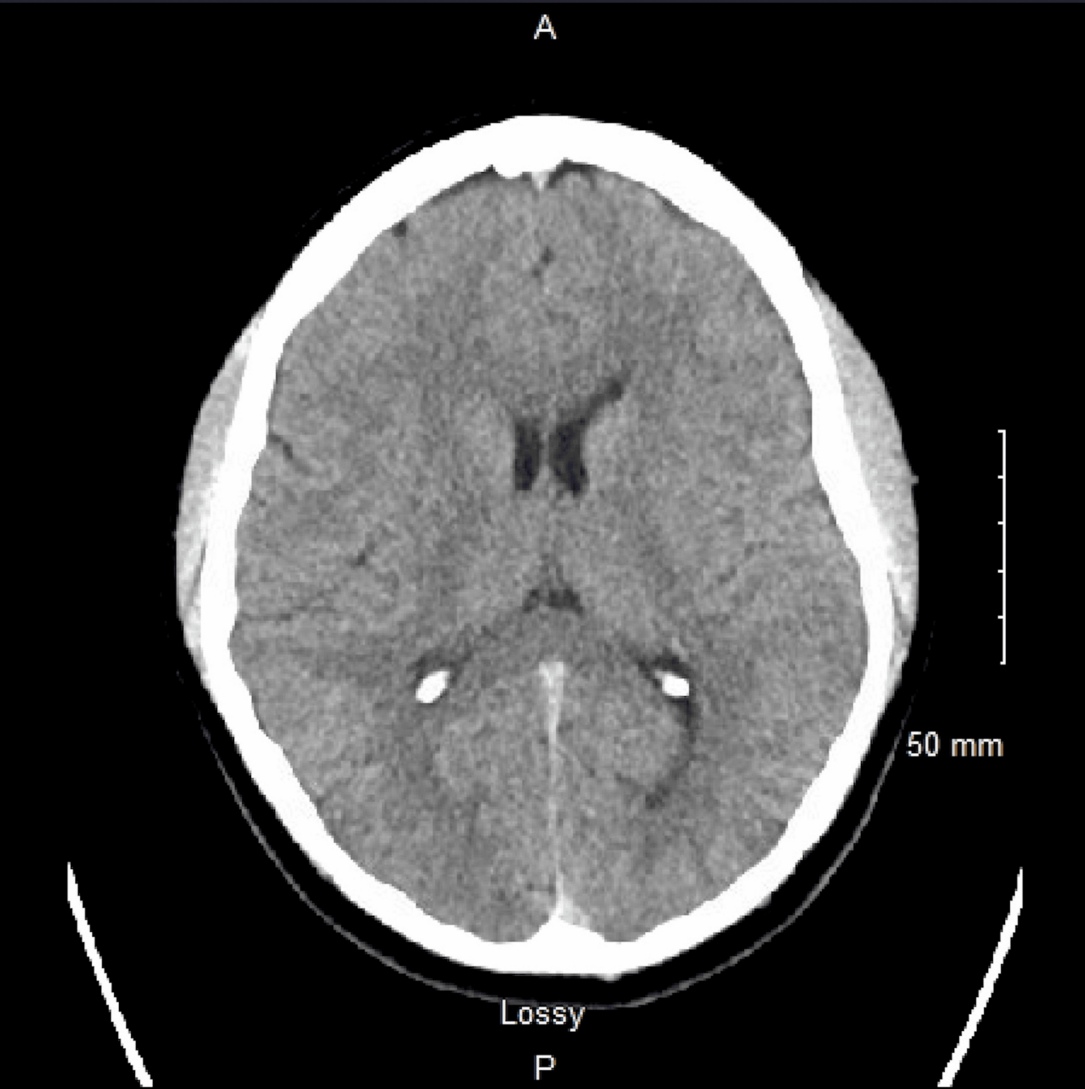
Head CT showing no acute abnormalities

The patient was a 37-year-old man with a past medical history of asthma that presented to the hospital due to seizures, confusion, and encephalopathy, beginning six hours prior to arrival via emergency medical services. The wife reported that the patient woke up confused and somnolent that morning, and just before they were going to leave for the hospital, he fell in the shower and she found him on the floor seizing. There were no symptoms prior to the episode except for a sore throat that was treated with oral Augmentin 500 mg that he was given at a local urgent care five days previously. Upon arrival, the patient was febrile (100.0°F, axillary) and tachycardic (108 beats per minute, regular) with normal respiratory rate (19 breaths per minute), O_2_ saturation (100%), and blood pressure (109/60 mmHg). The patient was not oriented to time, person, place, or situation. In the initial neurological physical examination, the patient had no focal neurological deficits, sensory deficits, or muscle weakness but was nonverbal and somnolent, likely due to being in a postictal state. The rest of the physical examination showed no abnormalities. Initial workup showed no metabolic derangements in his blood work (Table 1) besides mild hyperglycemia and hypermagnesemia, and CT scan of the head (Figure 1) revealed no acute ischemic or hemorrhagic injury. The neurology team was consulted and the patient received a loading dose with levetiracetam 3000 mg oral before being admitted to the step-down floor for close monitoring.

**Table 1:** Laboratory results at initial presentation WBC: White blood cell; RBC: Red blood cell; Hgb: Hemoglobin; Hct: Hematocrit; MCV: Mean corpuscular volume; MCH: Mean corpuscular hemoglobin; MCHC: Mean corpuscular hemoglobin concentration; RDW: Red cell distribution width; MPV: Mean platelet volume; ABG: Arterial blood gas; BUN: Blood urea nitrogen; GFR: Glomerular filtration rate; AST: Aspartate transferase; ALT: Alanine transaminase; TSH: Thyroid- stimulating hormone

Test	Lab Value	Normal Range
WBC	7.7 x10³/µL	5.0–11.0 x10³/µL
RBC	5.35 x10⁶/mm³	4.70–6.10 x10⁶/mm³
Hgb	15.1 g/dL	14.0–18.0 g/dL
Hct	44.5%	42.0–52.0%
MCV	83 fL	80–94 fL
MCH	28.2 pg	27.0–31.0 pg
MCHC	34.0 g/dL	33.0–37.0 g/dL
RDW coefficient of variation	13.8%	11.5–14.5%
Platelet count	243 x10³/µL	130–450 x10³/µL
MPV	7.9 fL	7.4–10.4 fL
Neutrophils % (Auto)	67.0%	42.0–75.0%
Lymphocytes % (Auto)	29.4%	20.0–50.0%
Monocytes % (Auto)	2.9%	0.0–9.0%
Eosinophils % (Auto)	0.2%	0.0–7.0%
Basophils % (Auto)	0.5%	0.0–2.0%
Neutrophils # (Auto)	5.2 x10³/µL	1.4–6.5 x10³/µL
Lymphocytes # (Auto)	2.3 x10³/µL	1.2–3.4 x10³/µL
Monocytes # (Auto)	0.2 x10³/µL	0.1–0.6 x10³/µL
Eosinophils # (Auto)	0.0 x10³/µL	0.0–0.7 x10³/µL
Basophils # (Auto)	0.0 x10³/µL	0.0–0.2 x10³/µL
Nucleated RBC % (Auto)	0.2%	0.0%
Patient temperature	99.9°C (likely °F)	36.1–37.2°C (97.9–100.4°F)
ABG pH	7.38	7.35–7.45
ABG pH (Temp Corrected)	7.373	7.350–7.450
ABG pCO_2_	42.3 mmHg	35.0–45.0 mmHg
ABG pCO_2_ (Temp Corrected)	43.6 mmHg	35.0–45.0 mmHg
ABG pO_2_	77.30 mmHg	75.0–100.0 mmHg
ABG pO_2_ (Temp Corrected)	81.00 mmHg	80.0–100.0 mmHg
ABG HCO_3_	25 mmol/L	22–26 mmol/L
ABG O_2_ sat (Measured)	95.7%	92.0–100.0%
ABG base excess	-0.5 mmol/L	-2.0–2.0 mmol/L
ABG oxyhemoglobin	95.30%	92.0–98.0%
Hgb (ABG)	15.900 g/dL	12.0–15.0 g/dL
Carboxyhemoglobin	0.1%	0.5–1.5%
Methemoglobin	0.3%	0.0–1.5%
Sodium	137 mmol/L	137–145 mmol/L
Potassium	4.1 mmol/L	3.4–5.0 mmol/L
Chloride	103 mmol/L	98–107 mmol/L
Carbon dioxide	20 mmol/L	22–30 mmol/L
Anion gap	13 mmol/L	10–20 mmol/L
BUN	8 mg/dL	9.0–20.0 mg/dL
Creatinine	0.90 mg/dL	0.66–1.25 mg/dL
Estimated creatinine clearance	118 mL/min	Not specified
Estimated GFR	113 mL/min/1.73m²	≥90 mL/min/1.73m²
BUN/Creatinine ratio	8.88	Not specified
Glucose	148 mg/dL	74.0–106.0 mg/dL
Calcium	9.3 mg/dL	8.4–10.2 mg/dL
Magnesium	2.40 mg/dL	1.60–2.30 mg/dL
Total bilirubin	0.50 mg/dL	0.20–1.30 mg/dL
AST	38 U/L	17–59 U/L
ALT	45 U/L	21–72 U/L
Alkaline phosphatase	62 U/L	38–126 U/L
Total creatine kinase	61 U/L	55–170 U/L
Troponin I	<0.012 ng/mL	0.012–0.034 ng/mL
Total protein	7.50 g/dL	6.30–8.20 g/dL
Albumin	4.6 g/dL	3.5–5.0 g/dL
Globulin	2.8 g/dL	2.0–3.9 g/dL
Albumin/Globulin ratio	1.7	Not specified
TSH (Reflex)	1.420 mIU/mL	0.465–4.680 mIU/mL
Salicylates	<1.0 mg/dL	0.0–20.0 mg/dL
Acetaminophen	<10.0 mcg/mL	10.0–30.0 mcg/mL
Serum alcohol	<10.0 mg/dL	<10.0 mg/dL

**Figure 2:**
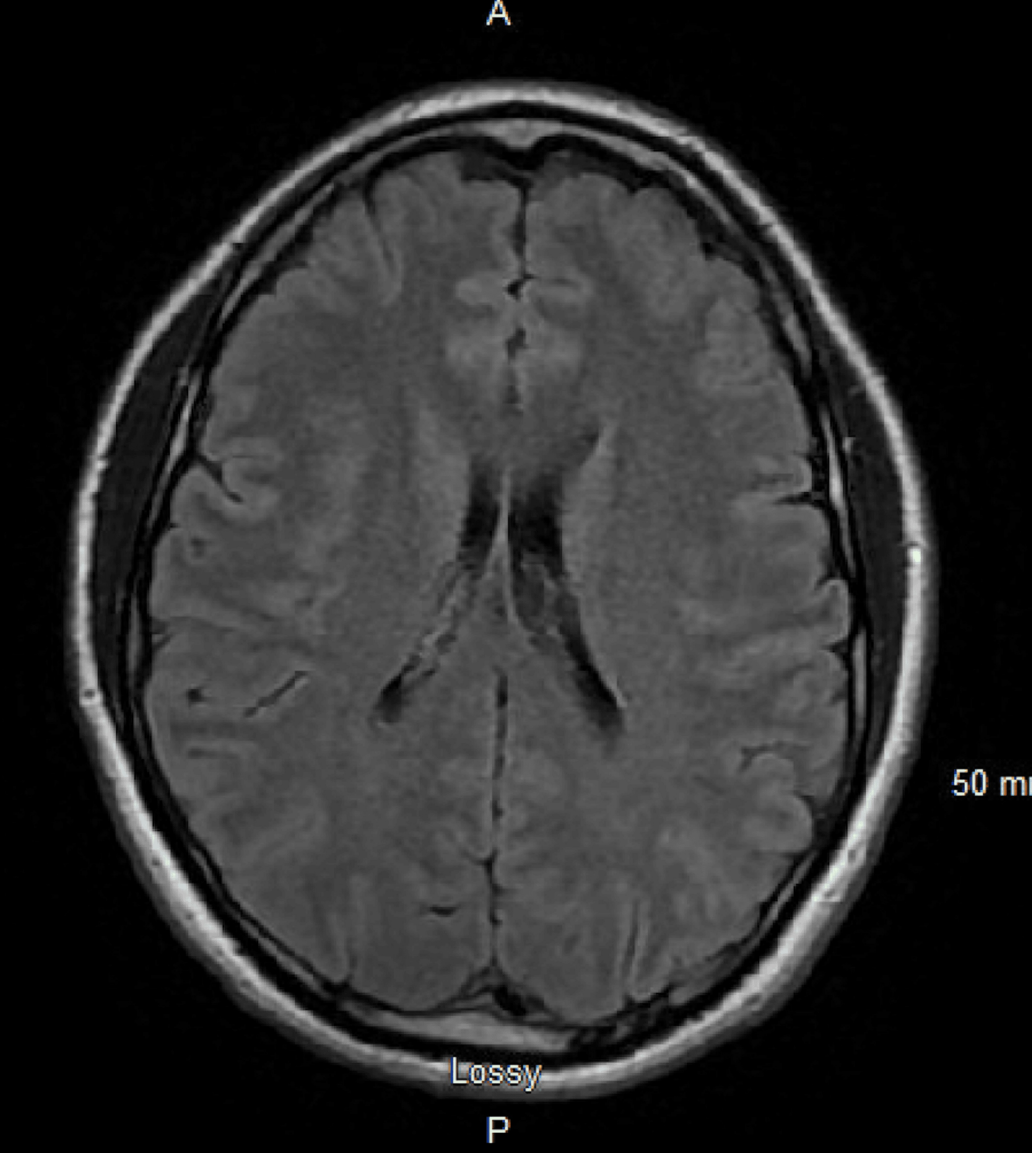
Head MRI showing no abnormalities

**Figure 3:**
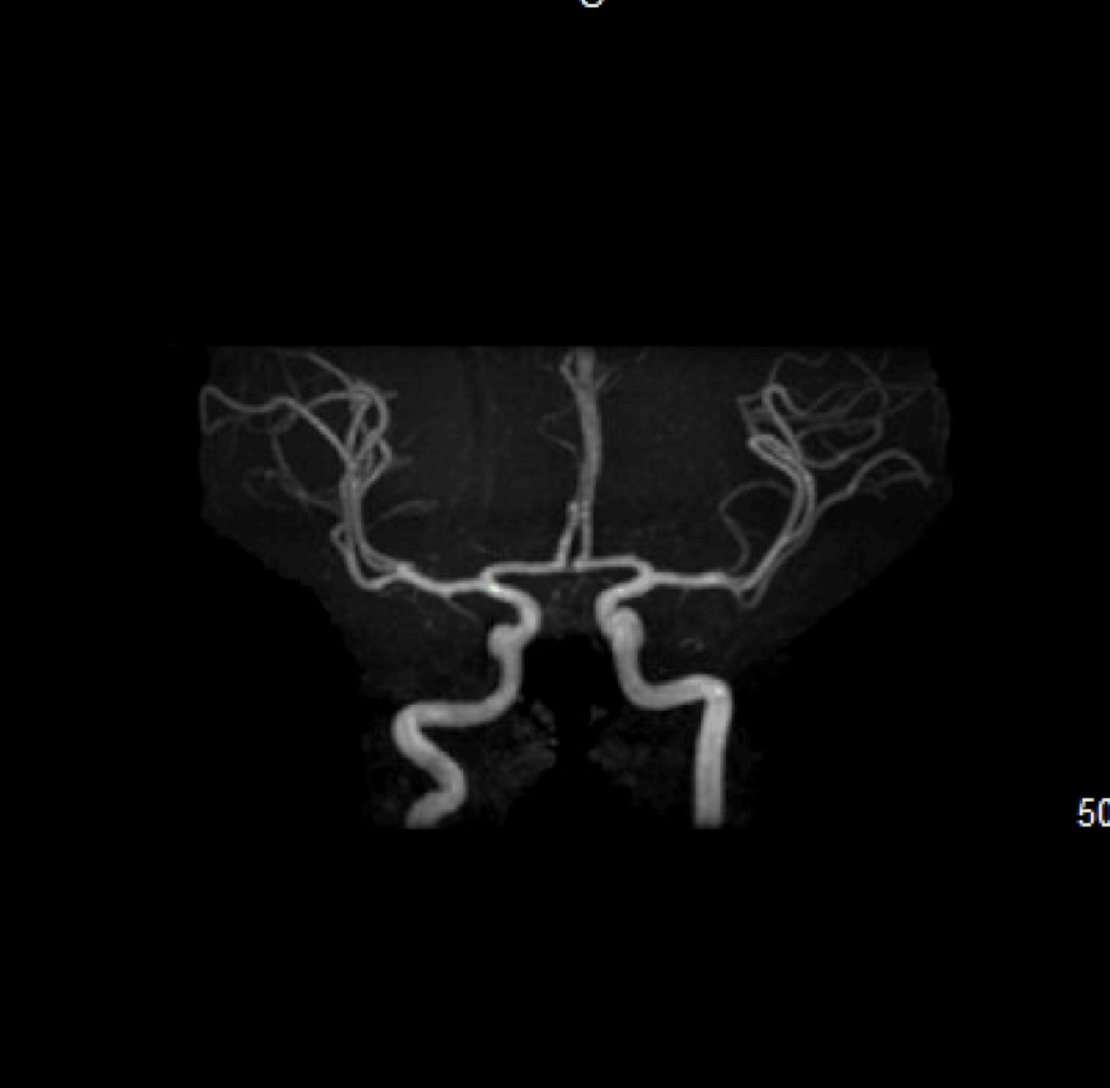
Head MRA showing no abnormalities MRA: Magnetic resonance angiography

**Figure 4:**
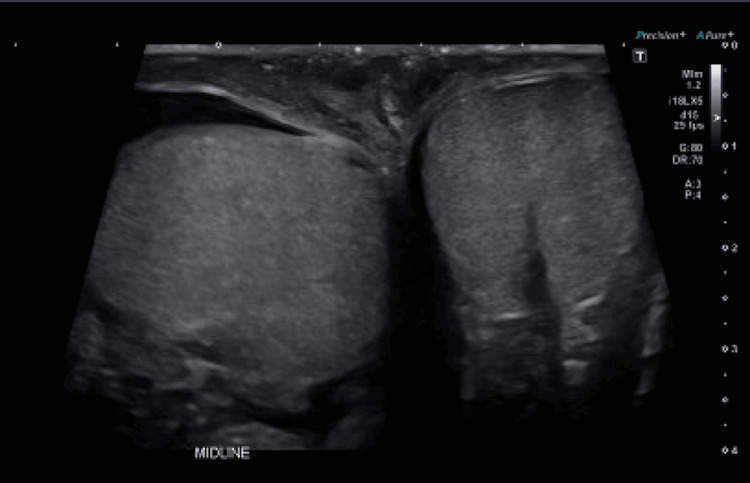
Testicular ultrasound showing no masses

Approximately 24 hours after admission, the patient had refractory seizures necessitating intubation for airway protection as his mental status deteriorated, and was placed in the ICU. Compared to the previous neurological exam on admission, the patient was more sedated and no longer able to follow commands. Seizure etiologies were explored: basic CSF panel was normal; CSF testing for bacterial, viral, and fungal etiologies was negative; urine drug screen was negative; MRI head with and without contrast (Figure 2) was negative; magnetic resonance angiography (MRA) head (Figure 3) was negative, and testicular ultrasound was negative for any mass (Figure 4), which could potentially lead to neoplastic limbic encephalitis, leading the neurology team to suspect autoimmune encephalitis, for which a serum panel was sent. Treatment began with IVIG 25 gm daily, followed by intravenous 1000 mg methylprednisolone daily.

While the autoimmune workup was pending, the patient continued to have multiple rounds of breakthrough seizures, and the decision was made to paralyze him with cisatracurium after it was noted that the status epilepticus was still ongoing despite being on four anti-seizure medications (2000 mg IV levetiracetam every 12 hours, 200 mg IV lacosamide every 12 hours, 300 mg topiramate via nasogastric tube every 12 hours, and 300 mg IV fosphenytoin twice daily). Status epilepticus persisted until an IV pentobarbital bolus 5 mg/kg over 15 minutes, followed by a drip of 3 mg/kg per hour, successfully controlled the seizures. The neurology team also started plasmapheresis for this patient due to failure of oral anti-seizure medications, and five rounds of infusions were completed.

While the patient remained sedated with intravenous ketamine 1000 mg, propofol 1000 mg, and midazolam 50 mg, plasmapheresis infusions continued and the patient was monitored for acute decompensation. Autoimmune encephalitis panel returned and was negative for all tested serum antibodies (Table 2). A new lab result on the same day returned a value of 249 for serum immunoglobulin G (IgG) antibody for *Mycoplasma pneumoniae*, which is indeterminate, but was followed up by a positive result for the serum IgM antibody for *Mycoplasma pneumoniae*. This finally suggested a possible cause for the seizures: *Mycoplasma*-induced seronegative autoimmune encephalitis. PCR was negative, implying a recent infection rather than an active one. Treatment was started with intravenous azithromycin 500 mg daily for seven days.

**Table 2:** Serum autoantibody testing results HNA: Human neutrophil antigen; Ab: Antibody; Meth: Method; Nuc: Nuclear; IgG: Immunoglobulin G; PCA: Purkinje cell cytoplasmic antibody

Test	Result
Anti-HNA Ab Meth	Negative
Neuronal Nuc Ab 2 (Ri)	Negative
Neuronal Nuc Ab (Hu/Ri)	Negative
Anti-Hu IgG Ab (IB)	Negative
Anti-Yo Antibody	Negative
PCA-1 (Yo) Ab	Negative

After approximately one month, the patient was successfully weaned off the sedative medications. He remained in the ICU for the remainder of his hospital course due to slow titration of his anti-seizure medications, which were at supratherapeutic doses. The rest of his hospital course was complicated with bilateral pulmonary emboli, greater on the right with possible right heart strain approximately one month after admission. The patient was also diagnosed with a *Pseudomonas* urinary tract infection and bacteremia with *Enterobacter cloacae* and *E. coli* approximately three months after admission, which were treated with IV ciprofloxacin 400 mg twice daily for seven days followed by oral ciprofloxacin 500 mg for seven days under infectious disease consultation. Lovenox was chosen for anticoagulation with plans for six months of treatment. Once the seizure medication regimen was carefully titrated, the patient was deemed ready for discharge with plans to follow up regularly with neurology in the outpatient setting.

## Discussion

This case illustrates a rare finding of *Mycoplasma pneumoniae*-induced seronegative autoimmune encephalitis by highlighting the challenges in diagnosis and management of this condition. While it is known that autoimmune encephalitis can cause seizures and encephalopathy, bacterial etiologies, particularly *Mycoplasma pneumoniae*, are infrequently documented in current literature. This case highlights the importance for clinicians to keep a high index of suspicion for *Mycoplasma*-associated autoimmune diseases even when autoimmune panels are initially negative.

In this case, multiple antiepileptic medications failed to alleviate the patient's refractory status epilepticus, necessitating the initiation of a medically induced coma. Further investigation into autoimmune explanations was necessary due to ongoing seizures and lack of response to conventional therapy. A positive IgM for *Mycoplasma pneumoniae* led the diagnosis toward an infectious trigger of an autoimmune process. This infectious cause of the patient’s encephalitis is supported by the limited knowledge currently available in the literature on *Mycoplasma*-associated neurological implications.

A few documented cases of *Mycoplasma pneumoniae*-associated autoimmune encephalitis reveal a spectrum of presentations including seizures, encephalopathy, and neuropsychiatric symptoms, often following respiratory prodromes. In one instance, a three-year-old boy presented with acute encephalopathy, irritability, and behavioral disturbances post* Mycoplasma* infection. He was treated with azithromycin and steroids, achieving full recovery without complications [6]. In another case, a 16-year-old girl with severe *Mycoplasma pneumonia* developed anti-IgLON5 antibody-associated encephalitis, with symptoms of cough, fever, and neuropsychiatric symptoms. She was managed with azithromycin, IVIG, steroids, and extracorporeal membrane oxygenation and had a near full recovery with mild cognitive decline at one year [7]. In one final case, a 40-year-old woman with mixed autoimmune encephalitis (anti-GAD65 encephalitis, Bickerstaff’s brainstem encephalitis, Hashimoto’s encephalopathy, Miller Fisher syndrome), post *Mycoplasma* infection, presented with seizures and respiratory failure. She was treated with IVIG, plasmapheresis, and steroids [8]. Our case’s prolonged ICU stay and bilateral pulmonary emboli were more severe than the documented cases, yet full recovery aligned with most reported cases, emphasizing aggressive therapy’s value.

While exact mechanisms are not known, it is proposed that immunological dysregulation and molecular mimicry, in which antibodies produced in response to *Mycoplasma* interact with neural tissues, are the mechanisms behind *Mycoplasma pneumoniae*-associated autoimmune encephalitis. Standard infection panels may return negative because this immunopathological process can take place without direct bacterial invasion of the central nervous system. Although there was no overt indication of a central nervous system infection in our patient, the existence of *Mycoplasma* IgM antibodies and clinical decline strongly points to an infectious autoimmune condition.

In order to minimize neuronal damage, early and aggressive immunosuppression is important in the treatment of autoimmune encephalitis. The patient in this instance got IVIG, plasmapheresis, high-dose steroids, several antiseizure drugs, and an antimicrobial medication. This patient’s eventual recovery after receiving immunotherapy and antibiotics highlights how important it is to treat both the infectious and autoimmune aspects of *Mycoplasma* infections.

This case presented multiple clinical complexities, including hospital-acquired infections and bilateral pulmonary emboli. The lack of a hypercoagulable state in other documented cases of *Mycoplasma pneumoniae*-associated autoimmune encephalitis implies that the pulmonary emboli were likely exacerbated by prolonged hospitalization rather than a hypercoagulable state induced by *Mycoplasma pneumoniae*, but the exact mechanism is unclear. Generally speaking, the long hospital course combined with immunosuppresion supports the notion that *Mycoplasma* infections may lead to multi-organ involvement and secondary complications.

This case illustrates the need for early detection and a comprehensive diagnostic workup, which includes testing for atypical infections, such as *Mycoplasma pneumoniae*, in patients with encephalopathy and refractory seizures. After ruling out other causes, serological testing for atypical organisms should be taken into consideration because autoimmune panels may come out negative in cases of *Mycoplasma*-induced encephalitis. Continued research is necessary to further understand the pathogenesis, diagnosis, and treatment regimens for this uncommon but potentially life-threatening condition.

## Conclusions

This case highlights the rare but significant association between *Mycoplasma pneumoniae* infection and seronegative autoimmune encephalitis, emphasizing the diagnostic and therapeutic challenges in such presentations. The patient’s course was marked by refractory status epilepticus requiring multiple antiepileptic medications, a medically induced coma, and intensive immunosuppressive therapy including IVIG, plasmapheresis, and high-dose corticosteroids. The delayed identification of *Mycoplasma pneumoniae* as the likely cause, despite extensive workup and initial negative autoimmune panels, underscores the need for clinicians to maintain a broad differential when evaluating unexplained encephalopathy. Additionally, the development of secondary complications related to the length of hospital stay reinforces the necessity of early recognition. Given the limited documentation of *Mycoplasma*-induced autoimmune encephalitis in the literature, further research is necessary to refine diagnostic criteria and establish standardized treatment protocols. Greater awareness of this condition can lead to earlier diagnoses, reduced neurological morbidity, and improved long-term recovery for affected patients. This case highlights the importance of considering *Mycoplasma pneumoniae *as a potential trigger for autoimmune encephalitis, especially in the absence of other identifiable causes. This association, although rare, broadens the spectrum of infectious agents linked to autoimmune-mediated neurological disorders.
